# Preferential production of G-CSF by a protein-like *Lactobacillus rhamnosus* GR-1 secretory factor through activating TLR2-dependent signaling events without activation of JNKs

**DOI:** 10.1186/s12866-015-0578-2

**Published:** 2015-10-26

**Authors:** Shahab Meshkibaf, Jӧrg Fritz, Marcelo Gottschalk, Sung Ouk Kim

**Affiliations:** Department of Microbiology and Immunology and Infectious Diseases Research Group, Siebens-Drake Research Institute, Western University, London, ON N6G 2 V4 Canada; Center for Human Immunology, Western University, London, ON N6G 2 V4 Canada; Department of Microbiology, McGill University, Montreal, QC H3G 0B1 Canada; Faculty of Veterinary Medicine, University of Montreal, St-Hyacinthe, QC J2S 2 M2 Canada

**Keywords:** *Lactobacillus rhamnosus* GR-1, G-CSF, Macrophage, TLR2, NF-κB, ERKs, Akt

## Abstract

**Background:**

Different species and strains of probiotic bacteria confer distinct immunological responses on immune cells. *Lactobacillus rhamnosus* GR-1 (GR-1) is a probiotic bacterial strain found in both the intestinal and urogenital tracts, and has immunomodulatory effects on several cell types including macrophages. However, detailed immunological responses and the signaling mechanism involved in the response are largely unknown.

**Results:**

We examined the production of GR-1-induced cytokines/chemokines and signaling events in macrophages. Among 84 cytokines and chemokines examined, GR-1 discretely induced granulocyte colony-stimulating factor (G-CSF) mRNA at highest levels (>60-fold) without inducing other cytokines such as IL-1α, IL-1β, IL-6 and TNF-α (<5-fold). The toll-like receptor (TLR) 2/6-agonist PAM_2_CSK_4_, TLR2/1-agonist PAM_3_CSK_4_ and TLR4-agonist lipopolysaccharide induced all of these inflammatory cytokines at high levels (>50-fold). The TLR2 ligand lipoteichoic acid activated all mitogen-activated kinases, Akt and NF-κB; whereas, GR-1 selectively activated extracellular regulated kinases and p38, NF-κB and Akt, but not c-Jun N-terminal kinases (JNKs) in a TLR2-dependent manner. Using specific inhibitors, we demonstrated that lack of JNKs activation by GR-1 caused inefficient production of pro-inflammatory cytokines but not G-CSF production. A secreted heat-labile protein-like molecule, 30–100 kDa in size, induced the preferential production of G-CSF.

**Conclusion:**

This study elucidated unique signaling events triggered by GR-1, resulting in selective production of the immunomodulatory cytokine G-CSF in macrophages.

**Electronic supplementary material:**

The online version of this article (doi:10.1186/s12866-015-0578-2) contains supplementary material, which is available to authorized users.

## Background

Microorganisms induce diverse immune responses, which can be harmful or beneficial to the host. Unlike pathogenic microbes, probiotics are microorganisms usually isolated from fermented food or healthy individuals, and confer a health benefit to the host when administered in adequate amounts [1, 2]. Among various probiotic bacteria, *Lactobacillus*, a Gram-positive facultative anaerobic bacterium, is a common constituent of the indigenous microbiota in the human intestinal and urogenital tracts [3, 4], and have been used as probiotics for preventing or treating infectious and inflammatory diseases [5, 6]. However, different *Lactobacillus* species and strains elicit strikingly different immune responses in a variety of immune cells and experimental systems [7–10]. *L. rhamnosus* GG (LGG) is a well-studied strain that can induce inflammatory responses in dendritic cells (DCs) and aggravate dextran sulfate sodium-induced acute colitis in mice [11, 12]. In contrast, LGG was shown to have anti-inflammatory effects on mouse and human macrophage cell lines [7, 13], and renders beneficial effects on chronic dextran sulfate sodium-induced mouse colitis and pouchitis in human [14, 15]. Two soluble factors from LGG, referred to as p75 and p40, prevent apoptotic cell death of intestinal epithelial cells, through activating the epithelial growth factor receptor [16, 17]. These factors ameliorate dextran sulfate sodium-induced acute colitis, as well as oxazolone and trinitrobenzenesulfonic acid-induced chronic colitis in mice [18]. GR-1, which is closely related to LGG, colonizes both the intestinal and urogenital tracts after oral supplements [19–21]. In human placental trophoblast cells, GR-1 increases IL-10 and G-CSF production, but suppresses TNF-α production [22, 23]. In the human bladder carcinoma cell line T24, GR-1 alone has little stimulatory effects in producing inflammatory cytokines and chemokines; however, potentiated *E. coli*-induced activation of the nuclear factor kappa-light-chain-enhancer of activated B cells (NF-κB) and production of inflammatory cytokines [24, 25]. GR-1 can also render anti-inflammatory effects on macrophages [26, 27] and dendritic cells [28], and promotes the generation of regulatory T cells in humans [29]. To date, detailed immune responses and signaling mechanisms elicited by GR-1 remain largely unknown.

Macrophages are key innate immune cells, orchestrating immune responses through releasing various pro- and anti-inflammatory cytokines. These cells are highly populated in the intestinal and urogenital tracts, interacting directly with microorganisms that have crossed the epithelial barrier [30, 31]. Macrophages detect microbe-associated molecular patterns (MAMPs) thorough pattern recognition receptors (PRRs) such as toll-like receptors (TLRs) and nucleotide-binding oligomerization domain (NOD)-like receptors. Although probiotics harbor MAMPs, they rarely cause infections and inflammatory diseases, but discretely modulate host immune responses through inducing distinct production profiles of cytokines and chemokines [7–10, 32]. Although details remain to be elucidated, several strains of probiotic bacteria have been shown to have immunomodulatory activities through selectively activating PRRs or regulating signaling cascades initiated by PRRs. For example, cell wall components such as peptidoglycan [33] and lipoteichoic acid (LTA) [34–36], cell wall associated polysaccharide [37], S layer protein A [38], bacteriocins [39, 40], pilus [41] and histamine [42, 43] have each been shown to modulate pro-inflammatory responses in macrophages and dendritic cells through activating specific or unidentified receptors. Also, several probiotic bacteria were shown to suppress inflammatory cytokine expression or promote anti-inflammatory cytokines by inhibiting activation of NF-κB [35, 44, 45], JNKs [46] and extracellular regulated protein kinases (ERKs) [35, 45]. The present study examined cytokines produced, signaling cascades activated, and bacterial factor(s) released by GR-1 in macrophages. We found that a protein-like factor released by GR-1 specifically and potently induced the immunomodulatory G-CSF through activating NF-κB, ERKs and Akt, but not JNKs, in a TLR2-dependent manner.

## Methods

### Mice

C57BL/6 J wild-type and TLR2^−/−^ (B6.129-Tlr2^tmlKir^/J) mice were obtained from The Jackson Laboratory (Bar Harbor, ME, USA) and were maintained in the animal care and veterinary facility at the Western University or University of Montreal, respectively. Nod2^−/−^ mice obtained from Dr. Jean-Pierre Hugot [47] were maintained in the animal care and veterinary facility at the McGill University. Male and female mice, aged 4–10 weeks, were used in all experimental procedures. All experimental protocols were approved by the Western University Animal Use Subcommittee, which follows the regulations of the Animals for Research Act (Ontario) and the Canadian Council on Animal Care.

### Cell cultures, bacteria and reagents

Bone marrow-derived immortalized macrophages from C57BL/6 J were generated as described previously [48] and cultured in RPMI 1640 medium (Sigma-Aldrich) supplemented with 10 % heat-inactivated fetal bovine serum (Sigma-Aldrich), 1000 U ml-1 penicillin, 10 mg ml-1 streptomycin (Sigma-Aldrich), 5 mM sodium pyruvate (Sigma-Aldrich), 5 mM MEM non-essential amino acids (Sigma-Aldrich) (referred to as complete RPMI; c-RPMI). Cells were then maintained at 37 °C in a humidified atmosphere with 5 % CO_2_. *L. rhamnosus* GR-1, obtained from Dr. Gregor Reid (The Canadian Research and Development Centre for Probiotics, Lawson Health Research Institute, London, ON, Canada), was grown anaerobically in De Man, Rogosa and Sharpe (MRS) agar (Becton Dickinson) using anaerobic packs (Becton Dickinson) at 37 °C for 48–72 h. For cell culture challenge, *L. rhamnosus* GR-1 were grown from a single colony in MRS broth (Becton Dickinson) at 37 °C for 24 h. Uropathogenic *E. coli* GR-12, originally isolated from the urine of a patient with pyelonephritis [49], and *Staphylococcus aureus strain* Newman were grown aerobically overnight in Luria-Bertani medium (Becton Dickinson) and brain-heart infusion broth (Becton Dickinson), respectively, with agitation at 37 °C. All bacteria were harvested by centrifugation at 6000 g for 10 min, washed three times with phosphate-buffered saline (PBS) (pH 7.4), and diluted to obtain a final optical density of 1.0 at 600 nm (representing approximately 10^9^ CFU/ml) in PBS. Lipopolysaccharide (LPS) from *E. coli* O111:B4 was from List Biological Laboratories (Campbell). PAM2 and PAM3 were purchased from Invivogen. LTA from *S. aureus* (indicated otherwise), lipase (from *Candida rugosa*), Cytochalasin D and RNase A were obtained from Sigma-Aldrich. DNase was purchased from Roche. LY294002, Akt inhibitor II, NF-κB activation inhibitor (CAS 545380-34-5), wortmannin, SB202190, U0126, and JNK inhibitor II were purchased from Calbiochem (EMD Biosciences). Antibodies for phospho-p38 (p-p38), p-ERKs, p-SAPK/JNKs, p-Akt, p-inhibitor κB (IκB) and β-actin were purchased from Cell Signaling Technology (NEB Biosciences).

### Generation of primary bone marrow-derived macrophages (BMDMs)

Bone marrow cells (BMCs) from wild-type, TLR2^−/−^, NOD1^−/−^, NOD2^−/−^, and corresponding cross-matched wild-type mice for each knockout mouse were harvested from femurs and tibia of mice using a 25.5-gauge needle and PBS. Isolated cells were cultured in c-RPMI supplemented with murine recombinant macrophage (M)-CSF (20 ng/ml; eBioscience). Cells were then maintained at 37 °C in a humidified atmosphere with 5 % CO_2_ for 7 days. The culture media were replaced with fresh media every two days after culture initiation.

### Crude LTA purification

Crude LTA was extracted from LGG and *S. aureus* using the butanol extraction method as previously described [50]. Briefly, bacterial cells were sonicated for 15 min, re-suspended in n-butanol/water (1:1, v/v) and stirred for 30 min at room temperature. The suspension was then centrifuged at 8000 g for 30 min, resulting in a two-phase system. The aqueous phase was lyophilized to give crude LTA. Subsequently, the lyophilized sample was re-suspended in PBS and used for further experiments.

### Macrophage cell challenge and cytokine determination

Macrophages were challenged in a 96-well plate format with 20 colony forming units (CFU/cell) of live bacteria, unless otherwise indicated, for 4 h in the presence of antibiotics-free media. Macrophages were then washed three times and further incubated with c-RPMI to prevent over-growth of bacteria and macrophage cell death. Macrophages were also treated with cell-free bacterial spent culture supernatant (SCS; 1/25 dilution), LTA (10 μg/ml) and LPS (100 ng/ml) in c-RPMI for the time indicated. Samples for TNF-α and G-CSF enzyme-linked immunosorbent assays (ELISA) were obtained from cell culture supernatant after 4 and 24 h challenges, respectively, unless otherwise indicated. Time points for TNF-α and G-CSF measurements were selected to maximize preferential production of G-CSF over TNF.

### ELISA

To measure the TNF-α and G-CSF levels in cell culture supernatant, ELISA kits were purchased from eBioscience (San Diego, CA) and R&D Systems (Minneapolis, MN), respectively. Standard curves were generated using recombinant proteins provided by the manufacturer.

### Western blot

Total cell lysate extraction and Western blot analysis were performed as previously described [26]. Briefly, total cell lysates were extracted using ice-cold lysis buffer containing 20 mM MOPS, 15 mM EGTA, 2 mM EDTA, 1 mM Na_3_VO_4_, 1 mM DTT, 75 mM β-glycerophosphate, 0.1 mM PMSF, 1 μg/mL aprotinin, 10 μg/mL pepstatin A, 1 μg/mL leupeptin, and 1 % Triton X-100. Following the incubation of cells with lysis buffer on ice for 5 min, cell lysates were extracted by centrifuging the homogenate at 18000 g for 15 min. Extracts were then mixed with SDS-PAGE loading buffer, heated to 100 °C for 5 min, resolved on 11 % SDS-PAGE polyacrylamide gels (Bio-Rad), and transferred onto nitrocellulose membranes. Following that, membranes were blocked with 5 % w/v skim milk for 30 min, immunoblotted with antibodies, and visualized using an enhanced chemiluminescence detection system (ECL; Pierce Bioscience). Band intensity quantification was performed using ImageJ software (National Institutes of Health, Bethesda, MD).

### Cytokines and Chemokines PCR Array

Total RNA was isolated using RNeasy Mini kit (QIAGEN Canada) according to the manufacture’s recommendations. Reverse transcription was conducted using the RT^2^ First Strand Kit (QIAGEN) according to the manufacture’s protocol. The Q-PCR was performed using the RT^2^ Profiler PCR Array System Kit for mouse cytokines and chemokines (QIAGEN) on an Applied Biosystems StepOnPlus instrument according to the manufacturer guidelines.

### Statistical analysis

Pooled results of several independent experiments were used in all analyses. Results are reported as mean ± SEM. Student’s *t*-test or one-way analysis of variance (ANOVA) with Tukey’s multiple comparison post hoc test were used to determine significance at *p* < 0.05 among experimental groups using Prism 5.0c for Mac OS X (GraphPad Software, La Jolla, CA).

## Results

### Production of G-CSF is the most prominent among 84 cytokines and chemokines examined in GR-1-treated macrophages

**Fig. 1:**
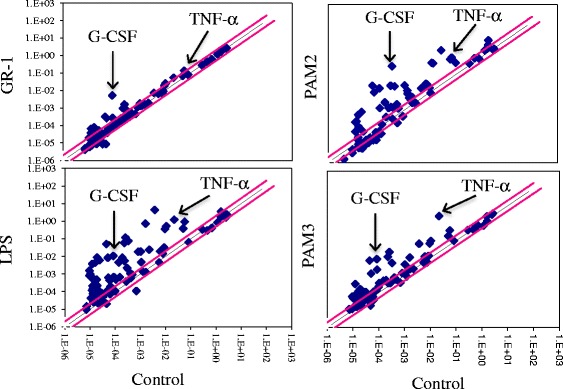
Production of G-CSF is the most prominent among 84 cytokines and chemokines examined in GR-1-treated macrophages. Immortalized BMDMs were treated with GR-1 (20 CFU/cell), lipopolysaccharide (LPS; 100 ng/ml), PAM_2_CSK_4_ (PAM2; 1 μg/ml) and PAM_3_CSK_4_ (PAM3; 1 μg/ml) for 5 h. mRNA expression of various cytokines and chemokines were measured using the RT^2^ Profiler PCR Array System Kit for mouse cytokines and chemokines. Data represent average of two independent experiments

We previously showed that GR-1 potently induces G-CSF but poorly induces TNF-α production in macrophages [28]. To further examine production of other cytokines induced by GR-1 and their levels in comparison to other stimuli, immortalized bone marrow-derived macrophages (BMDMs) were treated with live GR-1, LPS, PAM_2_CSK_4_ and PAM_3_CSK_4_ for 5 h, and the expression of 84 cytokines and chemokines were examined using the RT^2^ Profiler PCR Array System Kit. As shown in Fig. 1 (upper left panel) and Additional file 1: Table S1, GR-1 potently induced expression of G-CSF mRNAs (>60-fold of those induced in non-treated cells), whereas all others were induced less than 5-fold. LPS also induced G-CSF mRNA to ~80-fold; however, it also potently induced other cytokines and chemokines, including CCL5, CCL12, CXCL1, CXCL3, CXCL9, CXCL10, CXCL11, IL-1α/β, IL-6, IL-12, TNF-α and TNF superfamily member 10 more than 50-fold. PAM_2_CSK_4_ and PAM_3_CSK_4_ also potently induced G-CSF more than 100-fold; however, they also induced IL-1α/β, IL-16 and TNF-α more than 50-fold. These results demonstrate that GR-1 induced a cytokine/chemokine expression profile distinct from those of LPS, PAM2 and PAM3.

### Preferential G-CSF production by GR-1 is unique among other tested bacteria in primary BMDM

**Fig. 2:**
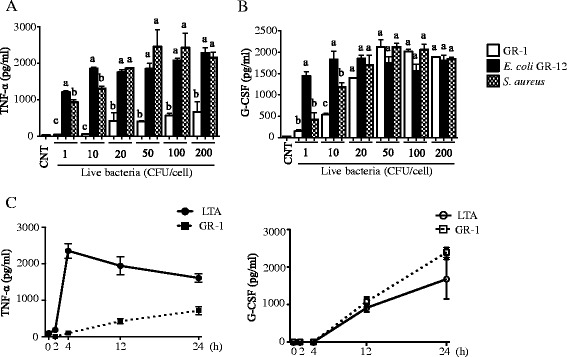
Preferential G-CSF production by GR-1 is unique among other bacteria in primary BMDMs. **a**-**b** Primary BMDMs were treated with live *S. aureus*, live *E. coli* GR-12 or live GR-1 at indicated bacteria/macrophage ratios (CFU/cell). Production of TNF-α (**a**; in 4 h) and G-CSF (**b**; in 24 h) was measured from cell culture media using ELISA. [*n* ≥ 3; *p* < 0.05 by one-way ANOVA with Tukey’s multiple comparison post hoc test; columns accompanied by the same letter (**a**, **b** or **c**) are not significantly different from each other]. **c** Primary BMDMs were treated with live GR-1 (20 CFU/cell) or lipoteichoic acid (LTA; 10 μg/ml) for indicated time points. Production of TNF-α (in 4 h) and G-CSF (in 24 h) was measured from cell culture media using ELISA. Data shown as mean ± SEM [*n* ≥ 3]

We further examined whether preferential production of G-CSF was also observed in other bacteria and also in non-transformed macrophages. BMDMs derived from C57BL/6j mice were treated with live GR-1, *E. coli* GR-12, and *S. aureus* with 1–200 CFU/cell, and production of TNF-α (in 4 h) and G-CSF (in 24 h) were measured using ELISA. Both *E. coli* and *S. aureus* induced TNF-α as low as 1 CFU/cell; whereas, GR-1 did not induce TNF-α even at 10 CFU/cell (Fig. 2a). GR-1 with higher than 20 CFU/cell induced TNF-α, but the levels was less than one-half of those induced by *E. coli* and *S. aureus*. Unlike TNF-α, G-CSF was significantly produced by all bacteria tested with similar maximal levels (Fig. 2b). GR-1 induced G-CSF production as low as 10 CFU/cell and maximally at 50 CFU/cell. *S. aureus* also gradually induced G-CSF production starting at 1 CFU/cell and maximally at 20 CFU/cell. *E. coli*, however, maximally produced G-CSF even at 1 CFU/cell. Collectively, these results suggest that *E. coli* and *S. aureus* induced both TNF-α and G-CSF in similar extents; whereas, GR-1 preferentially induced G-CSF over TNF-α. Further examination on the kinetics of the production of these cytokines showed that GR-1 induced G-CSF 12 h after treatment at similar levels induced by LTA (Fig. 2c, right panel). Unlike G-CSF, production of TNF-α was induced as early as 4 h of LTA treatment. However, GR-1 did not induce TNF-α until 12 h post-treatment and the levels were significantly lower than those by LTA (Fig. 2c, left panel). Similar results were also obtained in immortalized BMDMs. These results suggest that live GR-1 preferentially produced G-CSF with similar kinetics as LTA without prominently inducing TNF-α.

### Activation of ERKs, NF-κB and Akt but not JNKs by GR-1 plays a key role in the preferential production of G-CSF over TNF-α

**Fig. 3:**
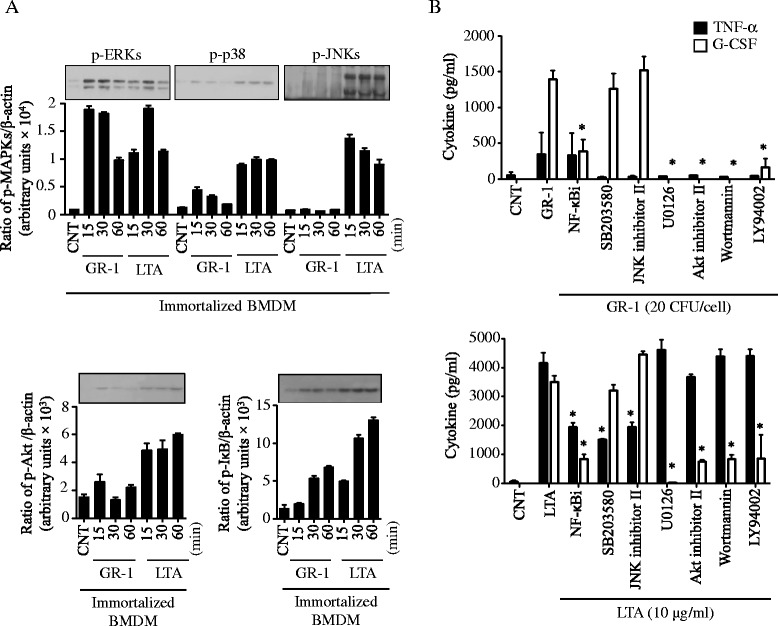
Activation of ERKs, NF-κB and Akt but not JNKs by GR-1 plays a key role in the preferential production of G-CSF over TNF-α. **a** Immortalized BMDMs were treated with live GR-1 (20 CFU/cell) or lipoteichoic acid (LTA; 10 μg/ml) for 15, 30 and 60 min. Phosphorylation of ERKs, p38, JNKs, Akt and IκB was analyzed by Western blots, using phospho-specific or β-actin antibodies. Bar graphs demonstrate band intensity quantification (ratio of protein phosphorylation to β-actin) using the NIH ImageJ program. Data shown as mean ± SEM [*n* ≥ 3]. **b** Immortalized BMDMs treated with GR-1 (20 CFU/cell) or lipoteichoic acid (LTA; 10 μg/ml) in the presence or absence of various inhibitors for NF-κB (NF-κBi; 10 μM), p38 (SB203580; 10 μM), JNKs (JNK inhibitor II; 250 nM), ERKs (U0126; 25 μM), Akt (Akt inhibitor II; 1 μM) and PI3K (wortmannin; 10 μM and PI3K-LY94002; 10 μM). Production of TNF-α (in 4 h) and G-CSF (in 24 h) was measured from cell culture media using ELISA. Data shown as mean ± SEM (*n* ≥ 3; *, *p* < 0.05 by Student’s *t*-test)

To examine signaling pathways activated by GR-1, immortalized BMDMs were treated with GR-1 at 20 CFU/cell for different time points and Western blots against phosphorylated IκB, Akt, and mitogen-activated protein kinases (MAPKs; ERKs, p38 and JNKs) were performed. Consistent with previous studies [51, 52], LTA activated all MAPKs, Akt and NF-κB signaling cascades after 15–30 min of treatment (Fig. 3a). GR-1 also potently activated ERKs, but weakly p38, Akt and NF-κB. However, no activation of JNKs by GR-1 was detected. To further examine which of these signaling cascades were involved in G-CSF and TNF-α production, these cells were pretreated with various inhibitors, and then treated with GR-1 or LTA. As shown in Fig. 3b, production of G-CSF by GR-1 and LTA was inhibited by the NF-κB inhibitor (NF-κBi), ERKs inhibitor (U0126), phosphatidylinositol 3-kinase (PI3K) inhibitor (wortmanin or LY94002) and Akt inhibitor (Akt inhibitor II), but not by the p38 inhibitor (SB203580) and JNKs inhibitor (JNK inhibitor II). Unlike G-CSF, the production of TNF-α by LTA was inhibited by all the MAPK inhibitors (U0126, SB203580 & JNK inhibitor II), but not by the PI3K and Akt inhibitors (wortmanin, LY94002 and Akt inhibitor II). These results suggest that activations of ERKs, NF-κB and Akt without activation of JNKs lead the preferential production of G-CSF over TNF-α in response to GR-1.

### TLR2 plays a key role in inducing G-CSF in GR-1-treated primary BMDM

**Fig. 4:**
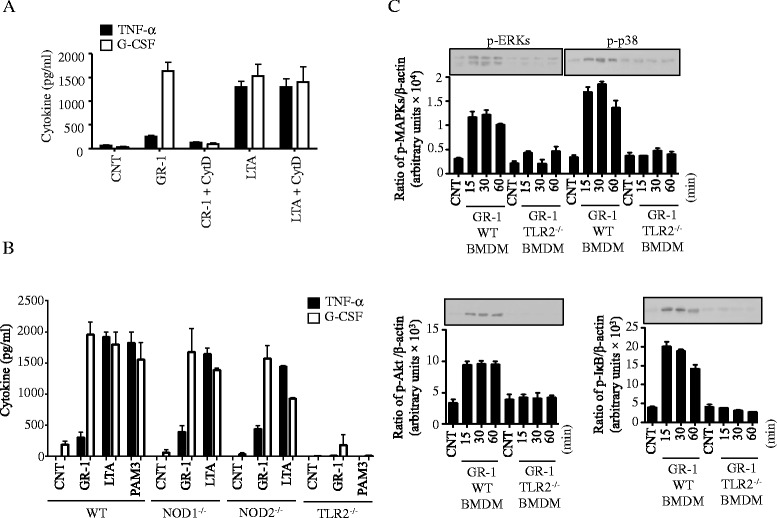
TLR2 plays a key role in inducing G-CSF in GR-1-treated BMDM in a phagocytosis dependent manner. **a** Immortalized BMDM were treated with the actin polymerase inhibitor cytochalasin D (CytD) for 1 h and then exposed to live GR-1 (20 CFU/cell) or lipoteichoic acid (LTA; 10 μg/ml). **b** Primary BMDMs prepared from wild-type (WT), TLR2^−/−^, NOD1^−/−^ and NOD2^−/−^ mice were treated with live GR-1 (20 CFU/cell), LTA (10 μg/ml) or Pam_3_CSK_4_ (PAM3; 1 μg/mL). **a**-**b** Production of TNF-α (in 4 h) and G-CSF (in 24 h) was measured from spent cell culture media using ELISA. Data shown as mean ± SEM [*n* ≥ 3]. **c** Primary BMDMs from wild-type (WT), TLR2^−/−^, NOD1^−/−^ and NOD2^−/−^ mice were treated with live GR-1 (20 CFU/cell) for 15, 30 and 60 min. Phosphorylation of MAPKs (ERKs, p38 and JNKs), Akt and I-κB was analyzed by Western blots, using phospho- or β-actin antibodies. Bar graphs demonstrate band intensity quantification (ratio of protein phosphorylation to β-actin) using the NIH ImageJ program. Data shown as mean ± SEM [*n* ≥ 3]

GR-1, as a Gram-positive bacterium, harbors several MAMPs, including lipoproteins/LTA and peptidoglycan, which activate TLR2 and NOD1/2, respectively. Also macrophages are professional phagocytes, and activation of these receptors and subsequent signaling events are coordinated by phagocytosis [53–55]. Thus, we examined if phagocytosis was involved in the preferential production of G-CSF, using the actin polymerization inhibitor cytochalasin D (CytD; 5 μg/ml). As shown in Fig. 4a, CytD pre-treatment inhibited G-CSF production induced by live GR-1 but not LTA, suggesting that phagocytosis of GR-1 was necessary for the production of G-CSF in macrophages. Since TLR2 and NOD1/2 are main receptors activated by Gram-positive bacteria, we then examined if these receptors are involved in GR-1-induced G-CSF production, using BMDM derived from TLR2-, NOD1- and NOD2-deficinent mice exposed to GR-1, PAM_3_CSK_4_ or LTA. G-CSF production was normal in NOD1- and NOD2-deficient, but undetectable in TLR2-deficient BMDMs in response to either GR-1 or PAM_3_CSK_4_ (Fig. 4b). NOD1- and NOD2-deficient BMDMs also produced similar levels of TNF-α as wild-type BMDMs by GR-1 and LTA, whereas TLR2-deficient BMDMs failed to produce TNF-α by either GR-1 or PAM_3_CSK_4_. Consistent with these results, TLR2-deficient primary BMDMs did not activate ERKs, p38, Akt and NF-κB in response to GR-1 (Fig. 4d). GR-1 activated JNKs in neither wild-type nor TLR2-deficient BMDMs (data not shown), as in immortalized BMDMs (Fig. 3a). These data suggest that production of G-CSF and activation of ERKS, p38, Akt and NF-κB by GR-1 was mediated through TLR2.

### A heat-labile protein-like molecule(s) secreted by GR-1 preferentially induces G-CSF production

**Fig. 5:**
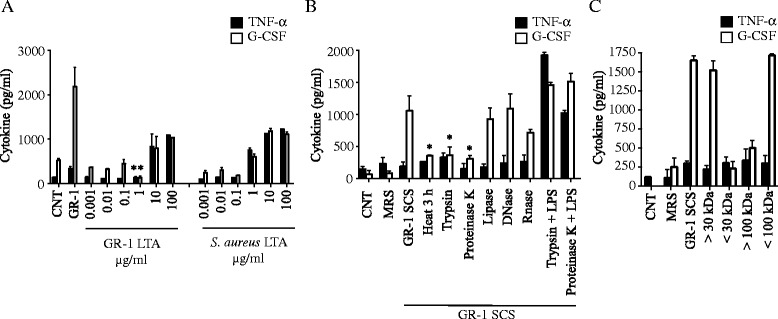
A heat-labile protein-like molecule(s) secreted by GR-1 preferentially induces G-CSF production. **a** Immortalized BMDMs were treated with indicated concentrations of GR-1- or *S. aureus-*n-butanol-extracted crude LTA. Production of TNF-α (in 4 h) and G-CSF (in 24 h) was measured from spent cell culture media using ELISA. Data shown as mean ± SEM (*n* ≥ 3; *, *p* < 0.05 by Student’s *t*-test). **b** Immortalized BMDMs were treated with GR-1-cell free spent culture supernatant (SCS; 1/25 dilution) or MRS culture media (1/25 dilution) which had been treated with proteinase K (200 μg/mL), lipase (200 μg/mL), trypsin (200 μg/mL), DNase (200 μg/mL), RNase (200 μg/mL), and heat (95^o^ C for 3 h). Production of TNF-α (in 4 h) and G-CSF (in 24 h) was measured from spent cell culture media using ELISA. Data shown as mean ± SEM (*n* ≥ 3; *, *p* < 0.05 by Student’s *t*-test). **c** Immortalized BMDMs were treated with MRS culture media (1/25 dilution), GR-1-SCS (1/25 dilution) or GR-1-SCS which had been fractioned using Centricon centrifugal filter devices with a 100 or 30 kDa molecular weight cutoffs. Production of TNF-α (in 4 h) and G-CSF (in 24 h) was measured from spent cell culture media using ELISA. Data shown as mean ± SEM [*n* ≥ 3]

Since TLR2 activation was required for G-CSF production by GR-1, we further examined whether crude LTA prepared from GR-1 induced a similar G-CSF preferential production effect in immortalized BMDMs. LTA from GR-1 was ~ 10-fold less effective in inducing both G-CSF and TNF-α than LTA prepared from *S. aureus* (Fig. 5a). However, unlike live GR-1, LTA from GR-1 induced both TNF-α and G-CSF at similar levels, suggesting that LTA was not the factor responsible for the preferential production of G-CSF. We then examined whether a factor(s) released from GR-1 had a similar effect on G-CSF production as in live GR-1. As shown in Fig. 5b, GR-1 cell free spent culture supernatant (SCS), but not the media (MRS), preferentially produced G-CSF over TNF-α, and the production of G-CSF was abolished in SCS treated with heat (95^o^ C for 3 h), trypsin and proteinase K, but not with lipase, DNase and RNase (Fig. 5b). Treatments of trypsin or proteinase K had no effects on LPS-induced TNF-α and G-CSF production, indicating that inhibition of G-CSF production in GR-1 SCS was not due to residual effects of these proteases on macrophages. To estimate the native molecular size(s) of the GR-1 molecule(s), GR-1-SCS was filtered through different membrane sizes. As shown in Fig. 5c, the molecule(s) passed through 100 kDa membranes but not 30 kDa membranes, suggesting that the GR-1 molecule(s) was between 100 and 30 kDa in native size. Collectively, these results indicate that the GR-1 factor(s) responsible for preferential G-CSF production in macrophages is a heat-labile protein-like molecule(s) of 30–100 kDa in native size.

## Discussion

Here, we showed that among 84 cytokines/chemokines examined, GR-1 uniquely and potently induced G-CSF (Fig. 1 and Additional file 1: Table S1), and the production of G-CSF by GR-1 was as efficient as other known pro-inflammatory stimuli in both kinetics and amounts (Fig. 2). This observation is consistent with our previous studies in macrophages [26, 28] and placental trophoblast cells that showed high levels of G-CSF production by GR-1 [22, 23]. GR-1 at higher than 20 CFU/cell or after 12 h post-exposure was able to induce TNF-α, albeit at significantly lower levels than those induced by *E. coli* GR-12 or *S. aureus* (Fig. 2). Since over-growth of GR-1 even after thorough wash and incubation with antibiotics containing cell culture media was detected, it is possible that the production of TNF-α in macrophages treated with GR-1 for 12–24 h could be due to high numbers of GR-1 (Fig. 2a and c). Nonetheless, GR-1 induced TNF-α much less efficiently when compared to other bacteria or LTA, which is consistent with previous studies shown in human bladder cells exposed to live GR-1 or GR-1 SCS [24, 25].

Inefficient production of TNF-α by GR-1 was in part due to poor stimulatory effects of GR-1 cell wall components. LTA prepared from GR-1 was ~10-fold less efficient in inducing TNF-α when compared to LTA similarly prepared from *S. aureus* (Fig. 5a). Previous studies have also shown that LTA purified from several strains of *Lactobacillus*, including *L. plantarum str.* WCFS1 [34], *L. casei str.* YIT9029 [35], *L. fermentum* YIT0159 [35] and LGG [56] are inefficient in producing pro-inflammatory cytokines such as TNF-α, IL-1β and IL-6. It is still unknown why LTAs from several strains of *Lactobacillus* are inefficient in inducing pro-inflammatory cytokines. Interestingly, mutant *L. plantarum* WCFS1 and LGG that lacks D-alanylated LTA induces more anti-inflammatory but less pro-inflammatory cytokines in monocytes than wild-type LTA [34], and rendered protective effects on colitis [12, 34]. However, in another study, alanylation of LTA had no role in the production of pro-inflammatory cytokines in LGG-treated macrophages and peripheral mononuclear cells [56]. Regardless the levels of alanylation, GR-1 LTA is not likely involved in the G-CSF preferential effect of GR-1, as GR-1 LTA at high doses induced both TNF-α and G-CSF at similar extents (Fig. 5a). Another cell wall component shown to be involved in immunomodulation by *Lactobacillus* is peptidoglycan [33, 57]. NOD1 and 2 are PRRs that recognize the peptidoglycan fragments γ-D-glutamyl-mesodiaminopimelic acid and muramyl dipeptides, respectively [58, 59]. However, GR-1 potently and preferentially induced G-CSF in macrophages deficient in either NOD1 or NOD2 (Fig. 4b), suggesting that GR-1 peptidoglycan is not involved in G-CSF preferential production by GR-1.

At this moment, the identity of GR-1 factor(s) responsible for the preferential production of G-CSF is elusive. This study suggested that a protein-like factor, which is sensitive to heat and proteases, such as trypsin and proteinase K, with a native molecular weight between 30 and 100 kDa, is responsible for G-CSF preferential production by GR-1 (Fig. 5b-c). To date, probiotic proteins known to preferentially induce anti-inflammatory cytokines include surface layer proteins from *L. reuteri*, *L. casei*, [60] and *L. acidophilus* NCFM [38], and bacteriocins released from *L. plantarum* strains [40, 61]. Immunomodulation elicited by surface layer proteins likely acts through DC-SIGN (DC-specific ICAM3-grabbing non-integrin) [60, 62], which is distinct from the GR-1 factor(s) that required TLR2 (Fig. 4a). Secreted factors of *L. rhamnosus* CNCM I-4036 and *Bifidobacterium breve* CNCM I-4035 were shown to be more potent than live bacteria counterpart in suppressing production of pro-inflammatory cytokines induced by *E. coli* or *Salmonella typhi*, respectively, in DCs [63, 64]. However, the identities of these factors are still unknown. Various *Lactobacillus* species, including *L. rhamnosus*, release bacteriocins which can directly modulate immune responses [61, 65, 66]. Therefore, bacteriocin-related proteins released by GR-1 may have been involved in the G-CSF production. Further studies on the possible involvement of bacteriocin-related proteins in G-CSF production are warranted.

GR-1 was shown to release factors that have immunomodulatory effects on non-immune cells. GR-1 SCS significantly induced G-CSF and macrophage inflammatory protein 1α/β, but suppressed various LPS-induced pro-inflammatory cytokines including TNF-α in human decidual cells [67] and in CD-1 pregnant mice [68]. However, in urinary bladder cells, GR-1 SCS enhanced activation of NF-κB [24] and production of TNF-α, but not IL-6 and IL-8, induced by *E. coli* [25]. In these studies, GR-1 or GR-1 SCS alone induced NF-κB but little TNF-α production, which is consistent with our results (Fig. 3a and 4b). Although it is unknown whether these responses are mediated by the same factor, molecular characteristics in a protein-like entity with native molecular size above 30 kDa appear to be common in both studies (Fig. 5) [24].

We further examined the signaling pathways required for G-CSF preferential production in GR-1-treated macrophages. GR-1 activated ERKs, p38, Akt and NF-κB, but not JNKs, in immortalized and primary BMDM in a TLR2-dependent manner (Fig. 3a and 4). Based on experiments using specific inhibitors, activation of ERKs, Akt and NF-κB was involved in G-CSF production, whereas activation of ERKs, p38, JNKs and NF-κB pathways was required for TNF-α production (Fig. 3b). These results are consistent with previous reports showing that p38 and JNKs are required for production of pro-inflammatory cytokines, such as TNF-α, IL-1β, IL-6 and IL-8 [69, 70]. Also, p38 and JNKs, but not ERKs, are involved in the signal transduction of LPS-induced TNF-α and IL-1β production by Kupffer cells [71] and RAW 264.7 macrophages [72]. Therefore, lack of JNKs activation in GR-1-treated macrophages likely contributed to the inefficient production of pro-inflammatory cytokines. The PI3K/Akt signaling axis can suppress TNF-α production through multiple pathways [73], particularly through inactivating NF-κB but activating cAMP response-binding protein (CREB), resulting in suppression of IL-12 and induction of IL-10 production [74]. We showed that the inhibitors specific for Akt or PI3K inhibited TNF-α, but not G-CSF, production (Fig. 3b). These results are consistent with previous studies showing that activation of ERKs, NF-κB and PI3K/Akt pathways are crucial for G-CSF expression [75, 76]. Recent studies also showed that activation of PI3K/Akt is required for TLR-mediated G-CSF production, through inducing expression of the transcription factor octamer-binding factor-2 (Oct-2) [77, 78]. Therefore, activation of PI3K/Akt and ERKs pathways by GR-1 is likely involved in the preferential production of G-CSF in GR-1-treated macrophages.

An intriguing fact is that G-CSF production by GR-1 required phagocytosis and TLR2 (Fig. 4a-b). We found that phagocytosis of GR-1 was required for efficient production of G-CSF (Fig. 4a). Recent studies have shown that TLR2 mediates signaling from endosomal vesicles, in addition to the plasma membrane, particularly during *Borrelia burgdorferi* infection [79, 80]. Therefore, it is possible that phagocytosis is required for the activation of endosomal TLR2 by a ligand(s) released or secreted from GR-1. Further studies are required to unravel whether GR-1 mainly activates endo/phagosomal TLR2. At this moment, it is also unknown how activation of TLR2 by GR-1 led to ERKs and p38 activation without JNKs activation, resulting in G-CSF production without inducing pro-inflammatory cytokines. However, the anti-inflammatory responses by activating TLR2 are not restricted in GR-1. *Bifidobacterium pseudocatenulatum* CECT7765 was recently shown to produce anti-inflammatory cytokines such as IL-10 and suppress TNF-α production through TLR2 [81]. Even purified LTA or whole cells from lactobacilli were previously shown to modulate TNF-α expression through a TLR2-dependent mechanism [34, 35, 82, 83]. Further experiments delineating mechanisms of TLR2 in activating immunomodulatory responses is of great interest.

The primary function of G-CSF in the generation and mobilization of neutrophils, G-CSF enhances anti-microbial function of mature neutrophils [84–86], and regulates immune responses through suppressing production of various pro-inflammatory cytokines in myeloid cells [26, 27], generating regulatory gut-homing macrophages [87], promoting maturation of tolerogenic DCs [28, 88, 89] and generation of IL-10-secreting CD4^+^ type 1 T regulatory cells [90]. Therefore, G-CSF production by GR-1 may play an important role in enhancing anti-bacterial activities of neutrophils and preventing cell death of intestinal/urogenital epithelial cells induced by various stresses, without inducing inflammatory responses. Beneficial effects of probiotics are not limited in the intestine. Interestingly, a recent study showed that administration of *L. plantarum* 299v or GR-1 protected cardiac failure and hypertrophy in the rat heart [91, 92]. Since G-CSF also renders cytoprotective effects against various stresses in neuronal and cardiac cells [93–95], we are tempted to speculate that G-CSF may also contribute to the cardio-protective effects of *L. plantarum* 299v or GR-1.

## Conclusion

In summary, this study demonstrated that a protein-like factor(s) from GR-1 preferentially and potently produced G-CSF in macrophages through selectively activating ERKs, NF-κB and Akt in a TLR2-dependent manner.

## Additional file

Additional file 1: Table S1. Expression of cytokines and chemokines in immortalized BMDMs treated with GR-1 and TLR ligands. (DOC 105 kb)

## Abbreviations

BMCs: Bone marrow cells
BMDM: Bone marrow-derived macrophages
CFU: Colony forming units
CytD: Cytochalasin D
DC: Dendritic cell
ECL: Enhanced chemiluminescence detection system
ELISA: Enzyme-linked immunosorbent assays
ERKs: Extracellular regulated protein kinases
G-CSF: Granulocyte colony-stimulating factor
GR-1: *Lactobacillus rhamnosus* GR-1
IL: Interleukin
IκB: inhibitory κB
JNKs: c-Jun N-terminal kinases
LGG: *Lactobacillus rhamnosus* GG
LPS: Lipopolysaccharide
LTA: Lipoteichoic acid
MAMP: Microbe-associated molecular pattern
MAPKs: Mitogen-activated protein kinases
NF-κB: Nuclear factor kappa-light-chain-enhancer of activated B cells
NOD: Nucleotide-binding oligomerization domain
PBS: Phosphate buffered saline
PI3K: Phosphatidylinositol 3-kinase
PRRs: Pattern recognition receptors
SCS: Spent culture supernatant
SDS-PAGE: Sodium dodecyl sulfate polyacrylamide gel electrophoresis
TLR: toll-like receptor
TNF-α: tumor necrosis factor-α
