# Supplementary material for: Preferential production of G-CSF by a protein-like Lactobacillus rhamnosus GR-1 secretory factor through activating TLR2-dependent signaling events without activation of JNKs
Source: BMC Microbiol. 2015 Oct 26;15:238. doi: 10.1186/s12866-015-0578-2 (PMC4623291; doi:10.1186/s12866-015-0578-2)
Supplement: Additional file 1: Table S1. — Expression of cytokines and chemokines in immortalized BMDMs treated with GR-1 and TLR ligands. (DOC 105 kb) [file 12866_2015_578_MOESM1_ESM.doc]

**Supplemental Table 1: Expression of cytokines and chemokines in immortalized BMDMs treated with GR-1 and TLR ligands.**

| Gene Symbol | Gene of Interest^1^ | Fold Change | | | |
| --- | --- | --- | --- | --- | --- |
|  |  | GR-1  (20 CFU/cell) | PAM2  (1 µg/ml) | PAM3  (1µg/ml) | LPS  (1 µg/ml) |
| Ccl1 | Chemokine (C-C motif) ligand 1 | N.D^2^ | N.D | N.D | 48 |
| Ccl12 | Chemokine (C-C motif) ligand 12 | 2 | 53 | 15 | 229 |
| Ccl19 | Chemokine (C-C motif) ligand 19 | -1 | -3 | 2 | 1 |
| Ccl2 | Chemokine (C-C motif) ligand 2 | 1 | 12 | 4 | 8 |
| Ccl22 | Chemokine (C-C motif) ligand 22 | -1 | 16 | 3 | 33 |
| Ccl24 | Chemokine (C-C motif) ligand 24 | -2 | -2 | -2 | -6 |
| Ccl3 | Chemokine (C-C motif) ligand 3 | 2 | 14 | 7 | 16 |
| Ccl4 | Chemokine (C-C motif) ligand 4 | 2 | 34 | 9 | 48 |
| Ccl5 | Chemokine (C-C motif) ligand 5 | -1 | 3 | 7 | 452 |
| Ccl7 | Chemokine (C-C motif) ligand 7 | 2 | 40 | 7 | 38 |
| Cd70 | CD70 antigen | N.D | N.D | N.D | 27 |
| Cntf | Ciliary neurotrophic factor | -1 | 1 | 1 | -1 |
| Csf1 | Colony stimulating factor 1 (macrophage) | -1 | 2 | -1 | 2 |
| Csf2 | Colony stimulating factor 2 (granulocyte-macrophage) | 3 | 12 | 24 | 8 |
| Csf3 | Colony stimulating factor 3 (granulocyte) | 61 | 161 | 142 | 124 |
| Cx3cl1 | Chemokine (C-X3-C motif) ligand 1 | -1 | 4 | 1 | 7 |
| Cxcl1 | Chemokine (C-X-C motif) ligand 1 | N.D | N.D | N.D | 85 |
| Cxcl10 | Chemokine (C-X-C motif) ligand 10 | 2 | 4 | 18 | 1183 |
| Cxcl11 | Chemokine (C-X-C motif) ligand 11 | N.D | N.D | N.D | 250 |
| Cxcl13 | Chemokine (C-X-C motif) ligand 13 | N.D | N.D | N.D | 11 |
| Cxcl16 | Chemokine (C-X-C motif) ligand 16 | 1 | 25 | 2 | 22 |
| Cxcl3 | Chemokine (C-X-C motif) ligand 3 | 5 | 155 | 24 | 163 |
| Cxcl9 | Chemokine (C-X-C motif) ligand 9 | N.D | N.D | N.D | 457 |
| Gpi1 | Glucose phosphate isomerase 1 | -1 | 2 | -1 | -2 |
| Il10 | Interleukin 10 | 4 | 5 | 2 | 24 |
| Il12a | Interleukin 12A | N.D | N.D | N.D | 159 |
| Il12b | Interleukin 12B | N.D | N.D | N.D | 269 |
| Il15 | Interleukin 15 | -1 | -1 | 1 | 11 |
| Il16 | Interleukin 16 | 1 | 3 | -1 | 6 |
| Il17f | Interleukin 17F | 1 | -1 | 1 | 1 |
| Il18 | Interleukin 18 | 1 | -1 | 2 | 4 |
| Il1a | Interleukin 1 alpha | 4 | 84 | 50 | 249 |
| Il1b | Interleukin 1 beta | 6 | 118 | 84 | 448 |
| Il1rn | Interleukin 1 receptor antagonist | 2 | 10 | 4 | 18 |
| Il23a | Interleukin 23, alpha subunit p19 | 1 | 1 | 3 | 5 |
| Il24 | Interleukin 24 | N.D | N.D | N.D | 11 |
| Il27 | Interleukin 27 | 1 | 5 | 3 | 43 |
| Il6 | Interleukin 6 | 7 | 214 | 154 | 1121 |
| Il7 | Interleukin 7 | N.D | -1 | N.D | 7 |
| Lif | Leukemia inhibitory factor | N.D | 34 | 26 | 45 |
| Lta | Lymphotoxin A | 1 | 2 | 3 | 4 |
| Ltb | Lymphotoxin B | 1 | 6 | 1 | 4 |
| Mif | Macrophage migration inhibitory factor | -1 | -1 | 1 | -2 |
| Osm | Oncostatin M | 1 | 4 | 2 | 3 |
| Pf4 | Platelet factor 4 | -1 | 5 | 1 | -1 |
| Ppbp | Pro-platelet basic protein | 1 | 4 | 2 | 3 |
| Spp1 | Secreted phosphoprotein 1 | 1 | 3 | 2 | 1 |
| Tgfb2 | Transforming growth factor, beta 2 | 1 | -3 | 2 | N.D |
| Tnf | Tumor necrosis factor | 2 | 90 | 114 | 156 |
| Tnfsf10 | Tumor necrosis factor (ligand) superfamily, member 10 | N.D | N.D | N.D | 298 |
| Tnfsf13b | Tumor necrosis factor (ligand) superfamily, member 13b | 1 | 2 | 1 | 2 |
| Vegfa | Vascular endothelial growth factor A | 1 | 4 | -1 | -1 |

^1^ Genes that were not detected in the PCR array by any of the treatments were not reported. For the complete list of genes refer to http://www.sabiosciences.com/ArrayList.php?pline=PCRArray (cytokines and chemokines PCR Array).

^2^ Genes that were not amplified or not detectable in the PCR array were marked as 'N.D' (not detected).
